# Hydrogen Reduction of Bauxite Residue for Green Steel and Sustainable Alumina Production

**DOI:** 10.1007/s40831-025-01046-x

**Published:** 2025-03-11

**Authors:** Manish Kumar Kar, Mengyi Zhu, Jafar Safarian

**Affiliations:** Department of Materials Science and Engineering, Norwegian University of Science and Technology, Alfred Getz Vei 2, 7491 Trondheim, Norway

**Keywords:** Bauxite residue, Hydrogen reduction, Cementing, Kinetics, Viscosity

## Abstract

This study introduces a novel approach in sustainable metallurgy for the efficient utilization and valorization of bauxite residue, aimed at producing sustainable alumina and green steel. The integrated process combines hydrogen reduction, alkaline leaching, and smelting of the leaching residue. Initially, the bauxite residue was pelletized with calcite and quicklime to create self-hardened pellets, leveraging the cementing effect of quicklime with water. These pellets underwent hydrogen reduction, achieving over 95% reduction, resulting in the formation of metallic iron and a leachable calcium aluminate phase for alumina recovery. The reduced pellets were then subjected to alkaline leaching, extracting 62% alumina. Subsequently, smelting at 1550 °C facilitated the near-complete separation of iron and calcium-rich slag. The process was analyzed using various analytical techniques, including X-ray diffraction, electron probe microanalysis, and inductively coupled plasma mass spectroscopy, complemented by thermodynamic calculations using FactSage 8.1 software. Iron oxide reduction to metallic iron was achieved at 1000 °C for 120 min, while sodium carbonate leaching effectively extracted alumina from the calcium aluminate slag. However, residual alumina was attributed to the formation of indissoluble gehlenite and a dense calcium carbonate layer that impeded leaching kinetics. Successful iron separation during smelting required temperatures above 1500 °C, though this process was challenged by the high viscosity of the oxide matrix and the purity of the iron.

**Graphical Abstract:**

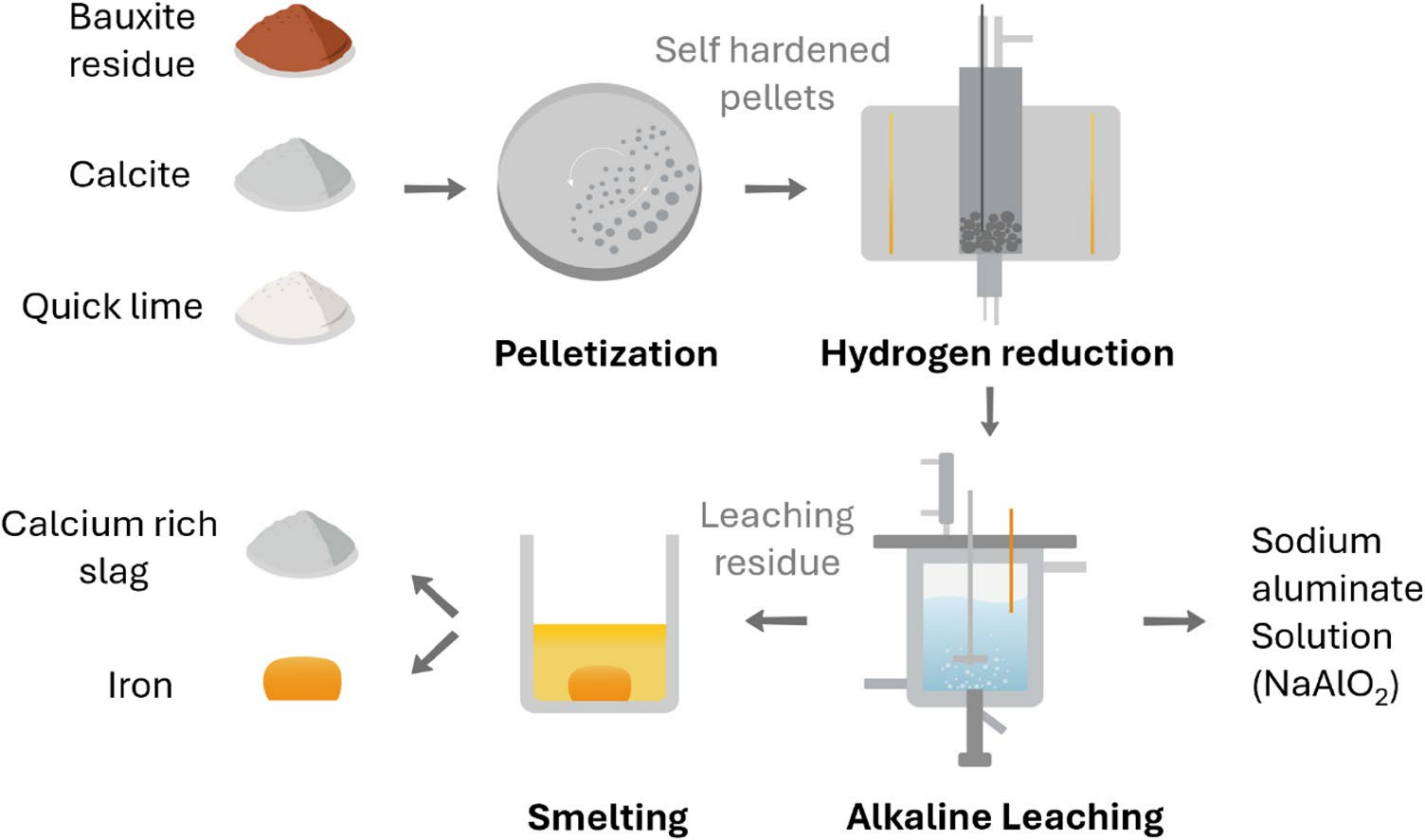

## Introduction

Globally, the Bayer process produces approximately 147.04 million tons of alumina annually in 2024 [1]. This process generates a hazardous by-product, red mud, with its dewatered form known as bauxite residue (BR). The quantity of BR generated depends on the processing conditions and the mineralogical composition of bauxite ore [2], with approximately 0.7–2.0 tonnes of BR produced per ton of alumina [3]. The global annual production of BR exceeds 150 million tonnes, and over 3.4–4.6 billion tonnes of BR have been stockpiled [4–6]. The industrial utilization of the generated BR faces considerable challenges due to its high alkalinity, fine particle size, and the presence of heavy metals. To date, only less than 3% of the total BR generation has been utilized in the construction industry, leaving the majority stored in dry heaps or tailing ponds [7–9].

In fact, BR contains dominant metal resources such as iron, aluminum, silicon, titanium, sodium, and calcium, along with rare earth elements in their oxide forms [4, 8, 10, 11], where iron oxide and alumina are the major constitutes by weight of the BR. Therefore, recovering iron and alumina from the residue can significantly reduce the volume of bauxite residue. Previous research has focused on recovering iron and alumina from BR through a carbothermic reduction process [12–14]. However, the carbothermic reduction process significantly contributes to greenhouse gas emissions by generating a significant amount of CO_2_. An alternative method involves replacing carbon with hydrogen as a reductant in place of carbon will generate water vapor in place of CO_2_/CO during reduction [15, 16]; nonetheless, the generation of hydrogen should come from renewable sources.

To date, only limited research has been conducted on the recovery of iron and alumina from the BR by using hydrogen as a reductant [11, 17–19]. Skibelid et.al. [18] studied the recovery of iron and alumina from BR by combined hydrogen reduction and alkaline leaching. In their study, BR pellets were sintered calcite at a high temperature of 1200 °C, followed by hydrogen reduction in a temperature range from 1000 to 1250 °C. They found that the reduction occurs more rapidly at 1000 °C compared to 1100 °C and 1200 °C, attributed to the loss of porosity at higher temperatures, which shifts the reduction mechanism toward being diffusion controlled. All iron-containing compounds were converted to metallic iron, and the alumina recovery exceeded 87% during alkaline leaching [18]. Stergi et al. [19] investigated the hydrogen reduction of BR with varying sodium hydroxide at different temperatures. They observed that with mass ratio 74/26 of BR and sodium hydroxide, the alumina recovery goes above 77%, and average iron content in the solid fraction was around 38.5%. The remaining solid phase mainly consisted of Ca, Si, and Ti, with a small fraction of undissolved Na and Al that less than 6 wt.% [19]. The main difference between Skibelid et al. [18] and Stergi et al. [19] lies in the alumina recovery method. Skibelid et al. utilize alkaline leaching of calcium aluminate slag, whereas Stergi et al. recover alumina through water leaching of sodium aluminate from bauxite residue in which another process is studied. However, both approaches share a similarity in the recovery of iron and alumina from bauxite residue.

Recent studies have focused on the iron recovery by magnetic separation from the hydrogen-reduced BR pellets [11, 20]. Iron separation was performed using medium-intensity magnetic separation, ranging from 1000 to 2500 gauss, and a Slon magnetic separator at 1000 gauss. It was found that as the magnetic intensity increased during magnetic separation from 1000 to 2500 gauss, the recovery improved for all particles sized in the medium-intensity magnetic separator. Particularly, the iron recovery enriched to 41% with the most appropriate particle size − 106 + 74 µm [21]. Hassanzadeh et al. [21] explored the iron recovery from BR-calcite reduced pellets by electrostatic separation and magnetic separation using the Davis tube and low-intensity magnetic separator. However, they discovered that while the electrostatic separation does not enhance the iron grade, there is an acceptable recoveries ranging from 22 to 37% received by Davis tube and low-intensity magnetic separator [11]. In a recent study, the recovery of iron and alumina from hydrogen-reduced BR-calcite pellets was conducted [22]. Initially, alkaline leaching by Na_2_CO_3_ solution was applied to recover alumina, followed by the magnetic separation of leaching residue to recover iron. It was found that alumina recovery goes above 75 wt.% and an enrichment of iron grade in the leaching residue to an acceptable range of 33 wt.% during magnetic separation was achieved [22]. In the physical separation studies mentioned above, the recovery is unsatisfactory, primarily attributed to the intricate structure of iron within the matrix.

This study presents a novel approach for the valorization of BR, with a focus on the extraction of iron and alumina as essential products to increase economic viability and environmental sustainability. The proposed BR recycling approach integrates hydrogen reduction, alkaline leaching, and smelting of leaching residue. By prioritizing the recovery of alumina through alkaline leaching, the process ensures a more efficient subsequent iron recovery by smelting with a reduced slag viscosity. This approach not only ensures the efficient extraction of metal resources but also improves the overall process efficiency, paving the way for sustainable waste management in the industry.

## Materials and Methods

**Fig. 1:**
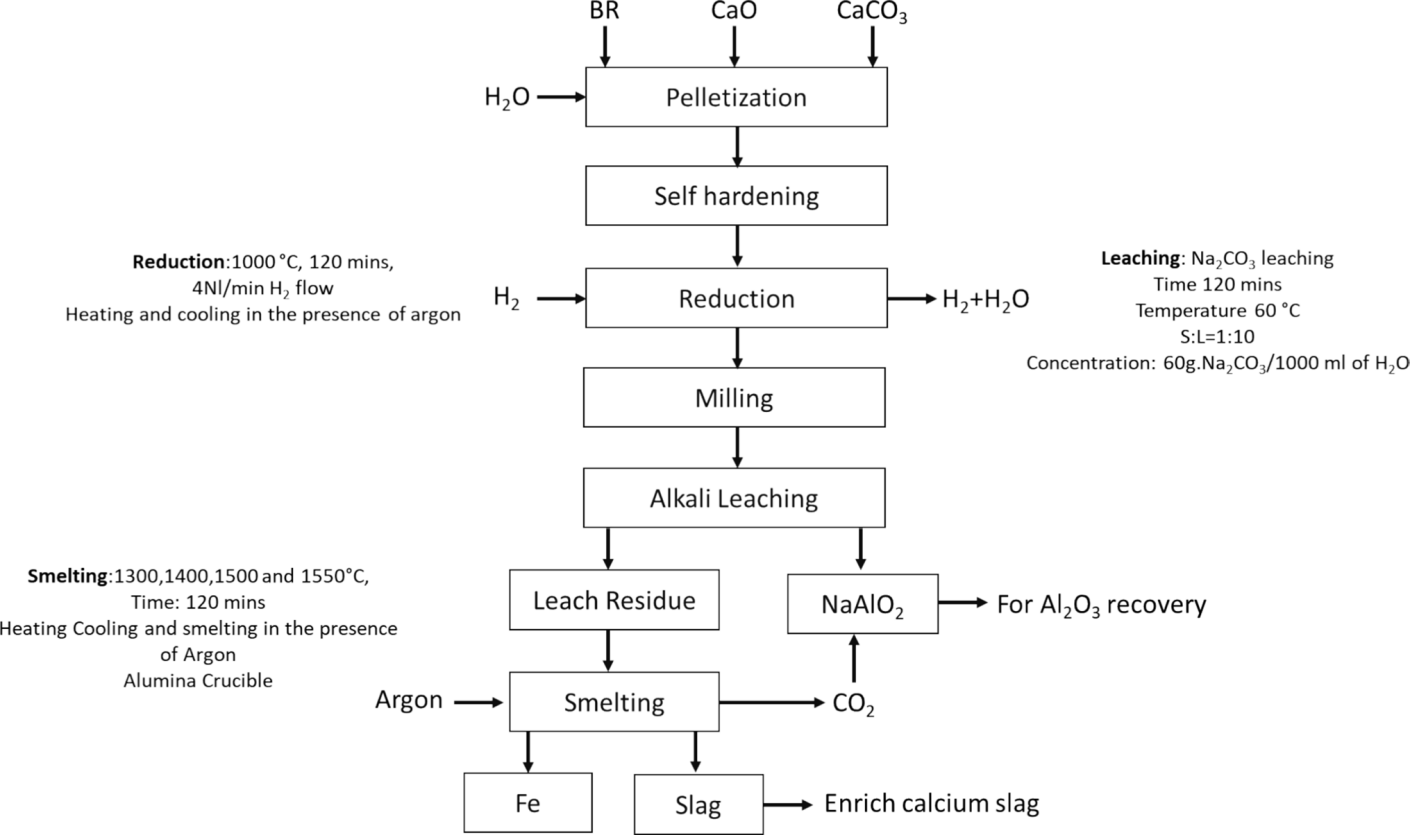
Overall flowsheet of the applied process

Figure 1 presents the process flowsheet applied in the present work for the BR valorization. The process begins by mixing BR with quicklime and calcite for pelletization with the addition of water. Subsequently, the pellets were self-hardened through aging over a period of few days [11, 23]. These self-hardened pellets were further subjected to hydrogen reduction. The resulting hydrogen-reduced pellets undergo further milling and alkaline leaching to recover alumina. Finally, the leaching residue was melted to recover iron and a calcium-rich slag.

### Materials

BR, limestone, and quick lime were supplied from Mytilineos S.A. (previously Aluminum of Greece), Vuglukli S.A. Greece, and NorFrakalk, respectively. These materials were received in lumpy form thus were deagglomerated and sieved to particle size of less than 500 µm. The deagglomerated BR, calcite, and quicklime were then mixed using a tabular mixture with specific ratios to from these phases CaO.Al_2_O_3_, 2CaO.SiO_2_, and CaO.TiO_2_ during the heat treatment process.

### Methods

#### Methods for Material Characterization

Phase analysis of the materials was carried out by X-ray diffraction (XRD) (Bruker D8 Focus, Bruker AXS GmbH, Karlsruhe, Germany) with CuKα radiation (wavelength *λ* = 1.54 Å). The diffractometer scans are in the range of 15° to 75° 2θ with a step size of 0.02°. The qualitative phase analysis of the raw data was analyzed by using DIFFRAC.EVA software with the database of PDF-4+ (2014, ICDD, Philadelphia, Pennsylvania, USA). To prepare the standard sample for XRD analysis, the materials were milled in WC vibratory disk mill (RS 200, RETSCH GmbH, Haan, Germany) for 45 s at an 800 revolution per minute (rpm). The microstructural analysis of the materials was done by electron probe microanalyzer (EPMA). The EPMA was supported with wavelength-dispersive spectroscopy (WDS) for elemental composition measurements.

#### Pelletization

The pelletization of mixed materials (BR, calcite, and quick lime) was conducted using a disk pelletizer via the addition of approximately 10 wt.% water. The composition of bauxite residue, calcite, and quicklime is mentioned elsewhere [22]. The pelletizer was operated at a revolution around 26 ± 2 revolution per minute (rpm) with a rotation angle of 45 degree, which was determined to be optimum angle for pelletization according to our previous work [24]. The size of the green pellets was around 5–10 mm. After pelletization, the green pellets were left in open atmosphere for 7 days to undergo self-hardening. The main purpose to do self-hardening is to increase the pellet's strength to tolerate further handling and reduction. The self-hardened pellets were dried in an oven at 80 ± 5 °C for 24 h to remove the remaining excess moisture of the pellets.

#### Reduction

**Fig. 2:**
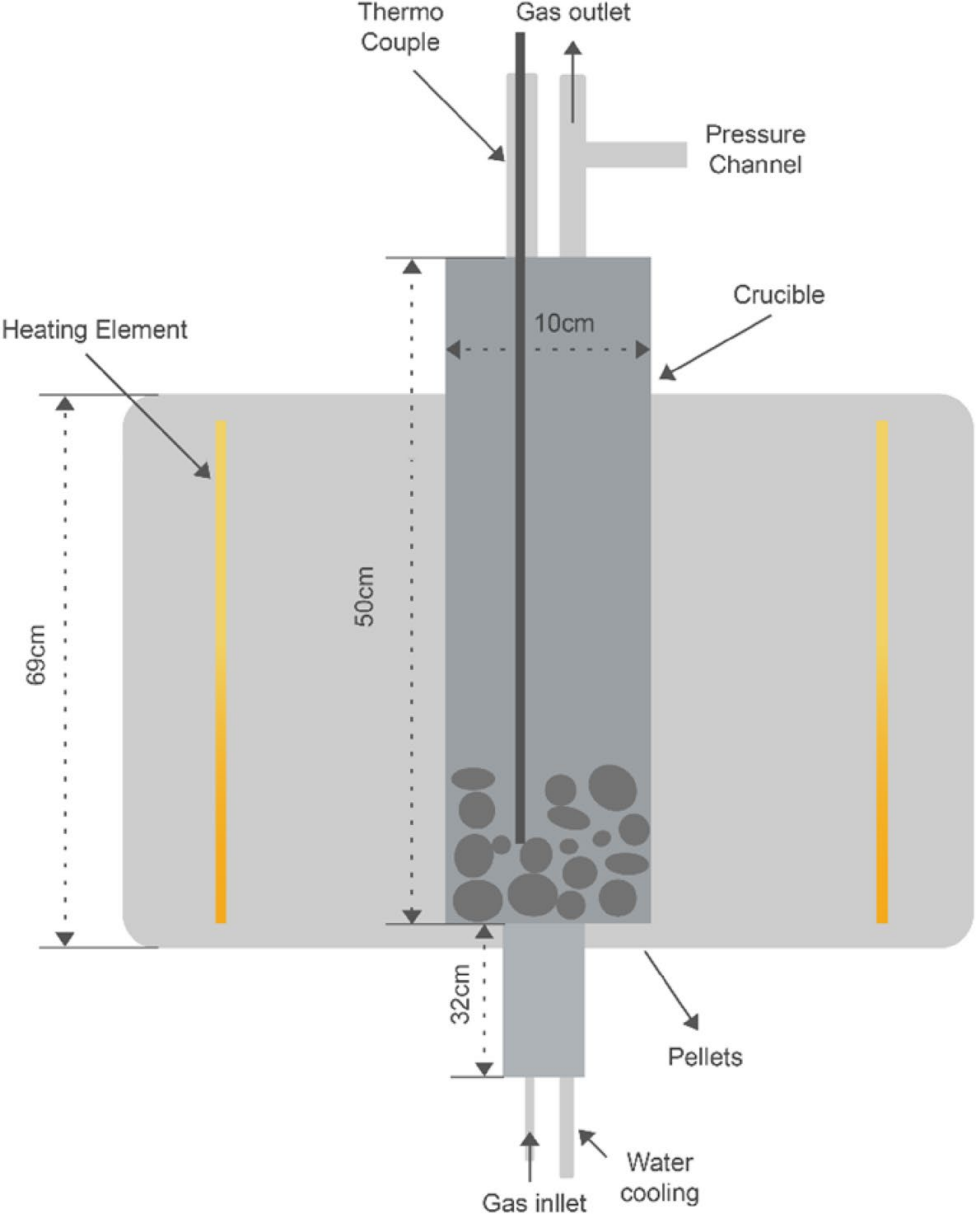
Schematic of vertical tube furnace used for hydrogen reduction

As illustrated in Fig. 2, the dried self-hardened pellets were reduced in a vertical tube furnace. Pellets were placed onto the crucible, which was then inserted vertically from the top of the furnace. Purging gases from the bottom of the crucible pass through the pellet bed before exiting via the off-gas outlet. Two thermocouples were used: one was inserted from the top into the pellet's bed, and the other one was on the furnace wall. The targeted temperature was set based on the two-thermocouple readings. The pellets were heated to the targeted temperature (1000 °C) in the presence of argon gas flow. Once the targeted temperature was reached, the gas flow was changed to hydrogen for 120 min with a flow rate of 4NL/min. The reduction time and temperature have been optimized based on our previous work [25]. To achieve a faster reduction rate and facilitate easier hydrogen recirculation, we used hydrogen with 100% purity. The furnace pressure was consistently maintained at 1 atmosphere.

After the completion of the reduction cycle, the hydrogen-reduced pellets were cooled under the purged argon stream to avoid reoxidation. The obtained reduced pellets were then grounded in a ball mill for 30 min with 25 rpm to achieve a particle size below 10 µm, ensuring an efficient leaching reaction.

#### Leaching

**Fig. 3:**
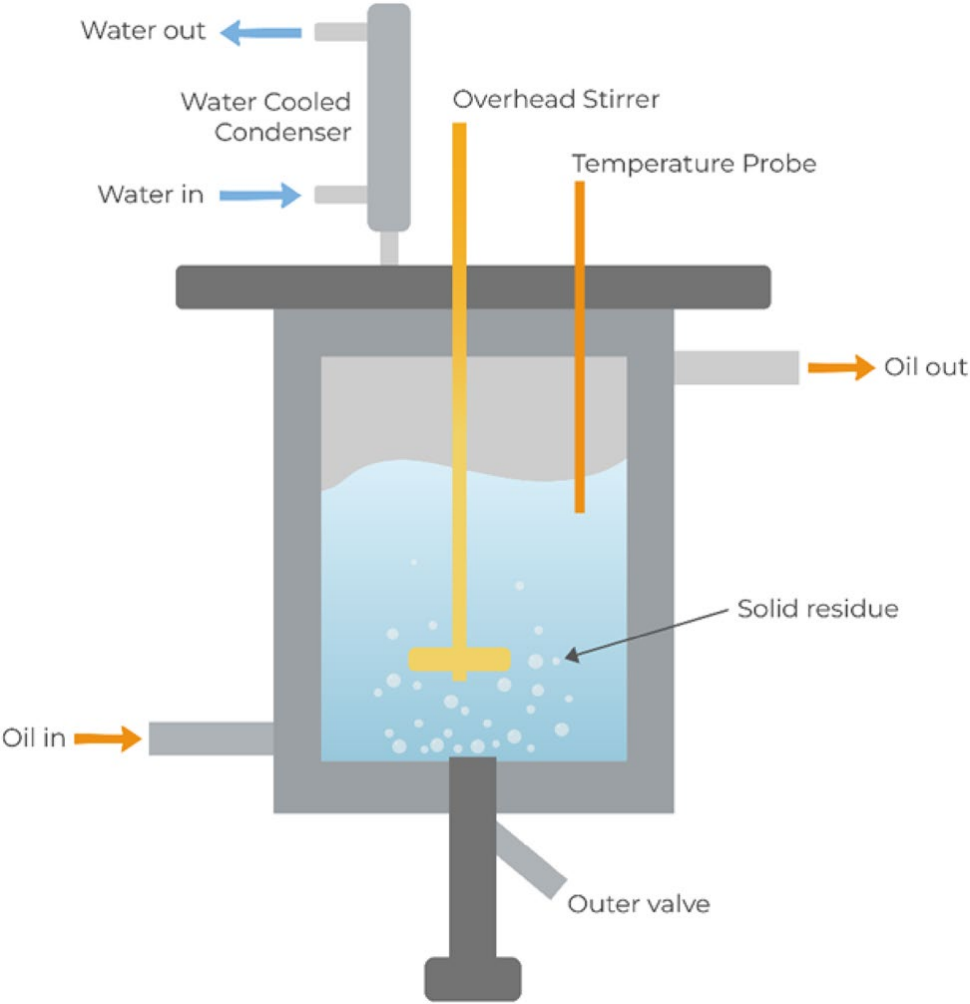
Schematic drawing of leaching reactor

The alkaline leaching of ground-reduced pellets was carried out in an oil jacketed glass reactor as shown in the Fig. 3. A mass of 100 g of ground-reduced pellets was added into a 1000 mL solution containing 60 g/L Na_2_CO_3_ which was heated to 60 °C via silicon oil circulated in a VMR thermobath. The temperature of the leaching solution was monitored by using a thermoprobe inserted from the top of the reactor, as depicted in Fig. 3. A shaft paddle impeller was also inserted from the top, the stirring speed was maintained at 400 rpm, and a condenser was also connected to the reactor to prevent water loss. The alkaline leaching period was held for 120 min. Upon completion of the leaching process, the slurry was filtered by using a vacuum pump with a 0.22-µm membrane. After filtration, the concentration of filtered liquor was measured by ICP-MS (inductively coupled plasma mass spectroscopy), while the leaching residue (dewatered gray mud) underwent XRD phase analysis.

#### Smelting of Gray Mud

After alumina recovery from the reduced pellets, the leaching residue predominantly composed of iron and calcite. It was melted to recover iron. The smelting of the leaching residue was conducted in a vertical tube furnace under argon purging, as illustrated and detailed in the operational description in our previous work [26]. The furnace temperature control was achieved through the use of two thermocouples: one positioned at the side of the furnace, and the other within the crucible from the top. The leaching residue was subjected to smelting at four different temperatures (1300, 1400, 1500, and 1550)  °C, each held for 120 min with argon gas purging. Above 1500 °C, there was complete separation of metallic iron from the rest of the oxide matrix.

## Results

### XRD Analysis

**Fig. 4:**
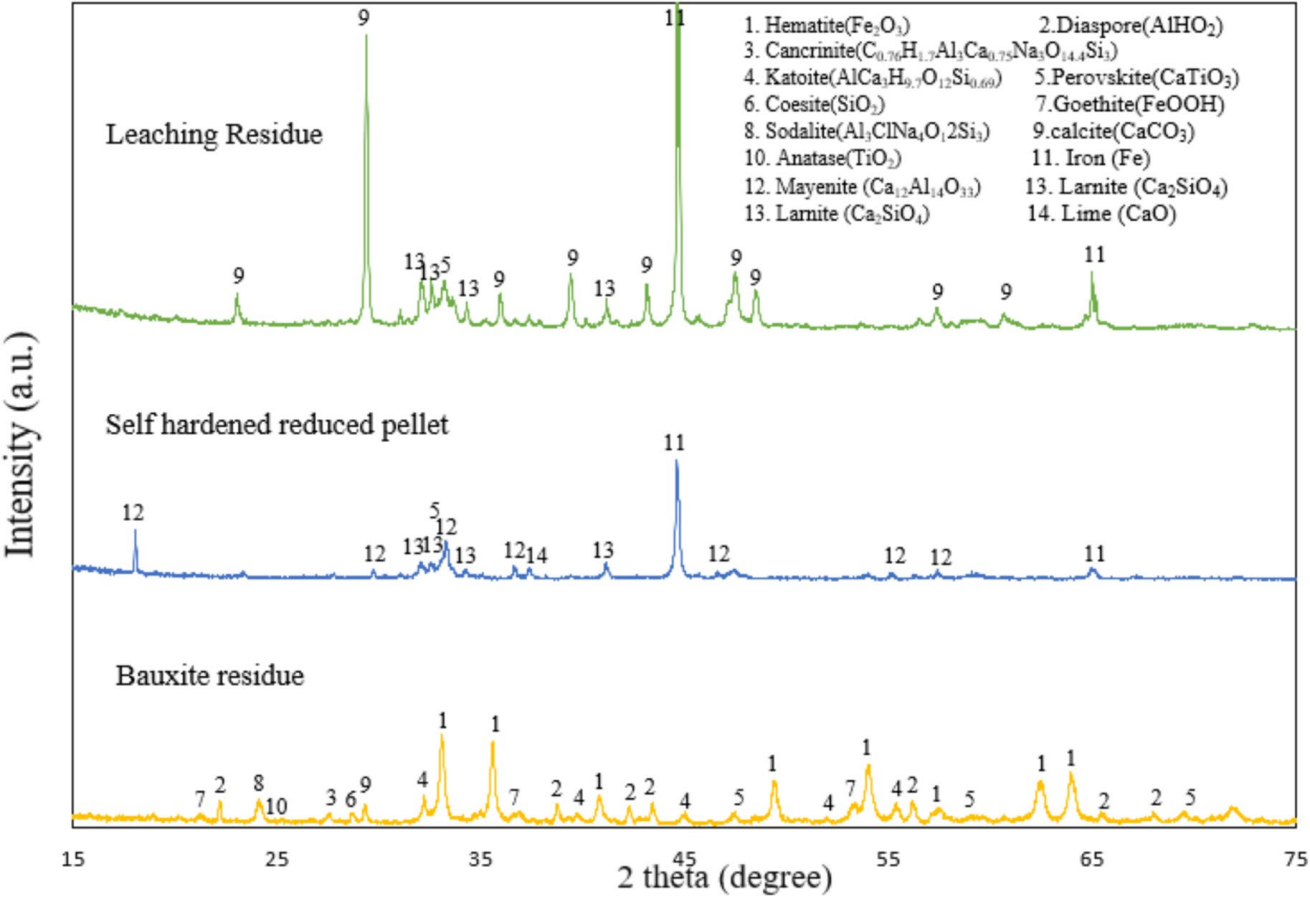
XRD spectra of bauxite residue, self-hardened reduced pellets, and leaching residue

The phase analysis of the bauxite residue, self-hardened reduced pellets, and leaching residue is presented in Fig. 4. In the bauxite residue, hematite (Fe_2_O_3_) and diaspore (AlHO_2_) are the major phase present. In the reduced pellets, identified phases correspond to metallic iron, mayenite (Ca_12_Al_14_O_33_), larnite (Ca_2_SiO_4_), lime (CaO), and perovskite (CaTiO_3_). Metallic iron and mayneite are the major peaks of the reduced pellets. However, in the leaching residue, iron and calcite emerge as the predominant peaks. The appearance of calcite in the leaching residue is due to its formation during the alkaline leaching of mayneite as nearly all the mayenite phase was reacted.

**Fig. 5:**
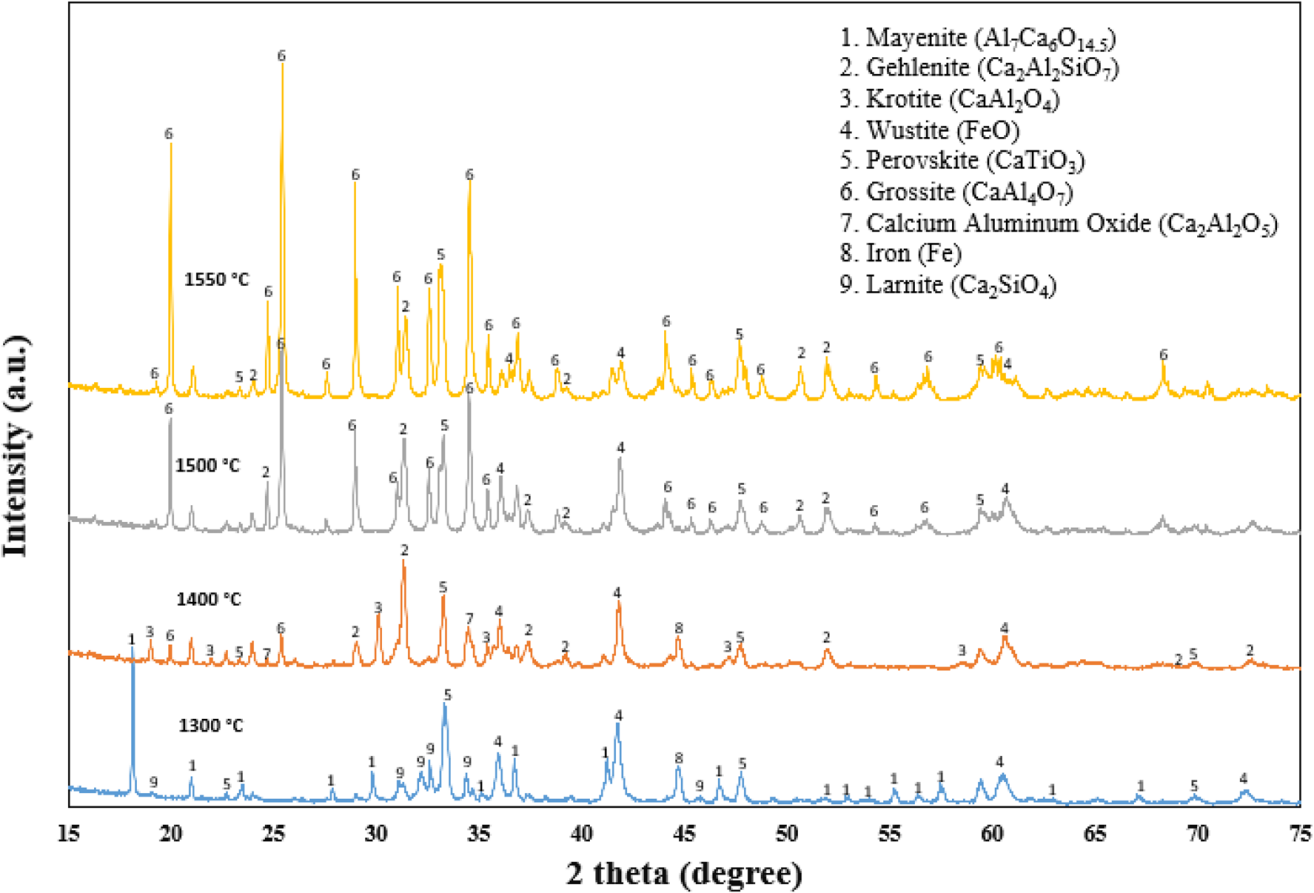
Phase analysis of the smelted leaching residue at different smelting temperatures

Figure 5 shows the phase analysis of the smelted leaching residue at different temperatures. The major phases identified in the smelted slags are mayenite (Ca_12_Al_14_O_33_), gehlenite (Ca_2_Al_2_SiO_7_), krotite (CaAl_2_O_4_), wüstite (FeO), perovskite (CaTiO_3_), grossite (CaAl_4_O_7_), dicalcium aluminate (Ca_2_Al_2_O_5_), and metallic iron (Fe). The slag smelted at 1300 °C shows the existence of mayenite, perovskite, and wüstite. Additionally, the presence of metallic iron was found along with those phases. However, with increasing the smelting temperature, grossite (CaAl_4_O_7_) becomes more stable. In all the smelted slags, a portion of wüstite (FeO) is present, with its amount decreasing at higher temperatures. However, the majority of metallic iron separates from the oxide matrix at temperatures exceeding 1500 °C. Additionally, a minor fraction of krotite is observed at temperatures above 1400 °C.

### Microstructural Analysis

**Fig. 6:**
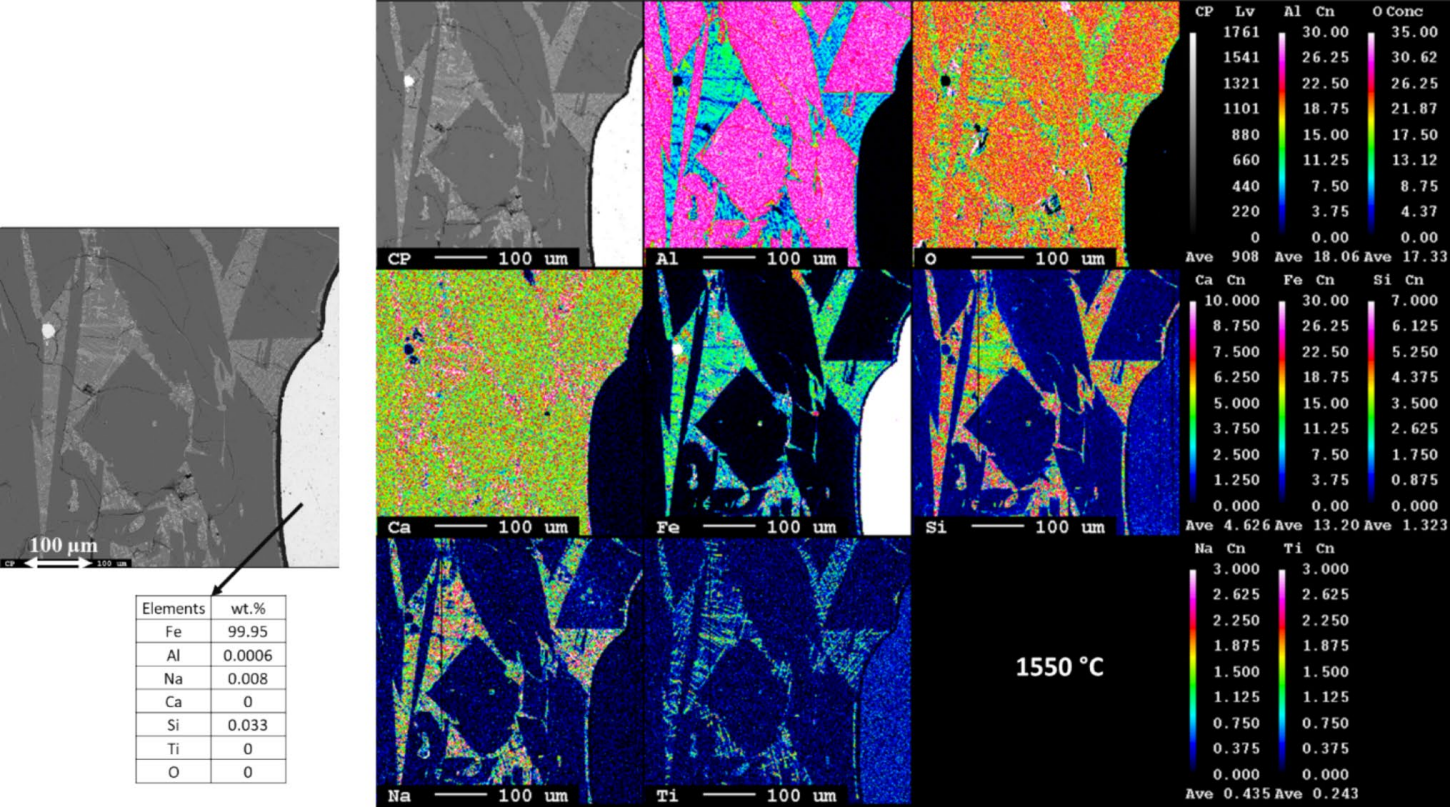
Microstructure and elemental mapping of smelted leaching residue at 1550 °C

The microstructure and elemental mapping of the metallic iron and slag of 1550 °C are shown in Fig. 6. The bright area represents the metallic iron phase, where iron is the predominant element with only very little fraction of Al and Na impurities. In the slag, two major phases are observed: the dark phase primarily composed of Al, O, and Ca, likely corresponding to calcium dealuminate (CaAl_4_O_7_), as correlated to the form XRD phase analysis, and the lighter dark slag phase is a composite mixture of various elements where Al, Fe, O, Na, Ca, and Ti are present. This region possibly comprises a composite of phases such as perovskite, gehlenite, and wüstite. In addition, Fig. 6 also indicates a complete separation of iron from the slag.

**Fig. 7:**
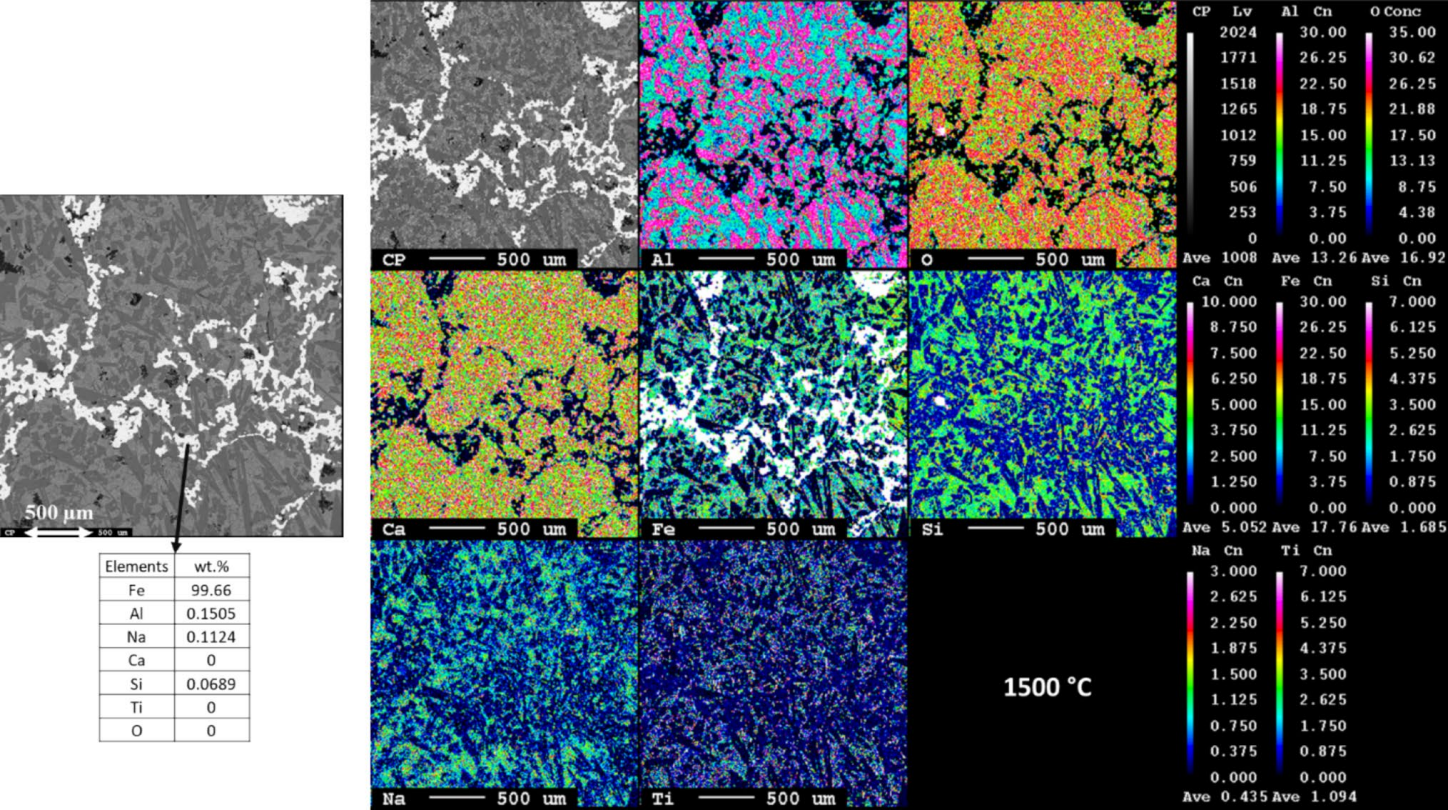
Microstructural and elemental mapping of leaching residue slag smelted at 1500 °C

**Fig. 8:**
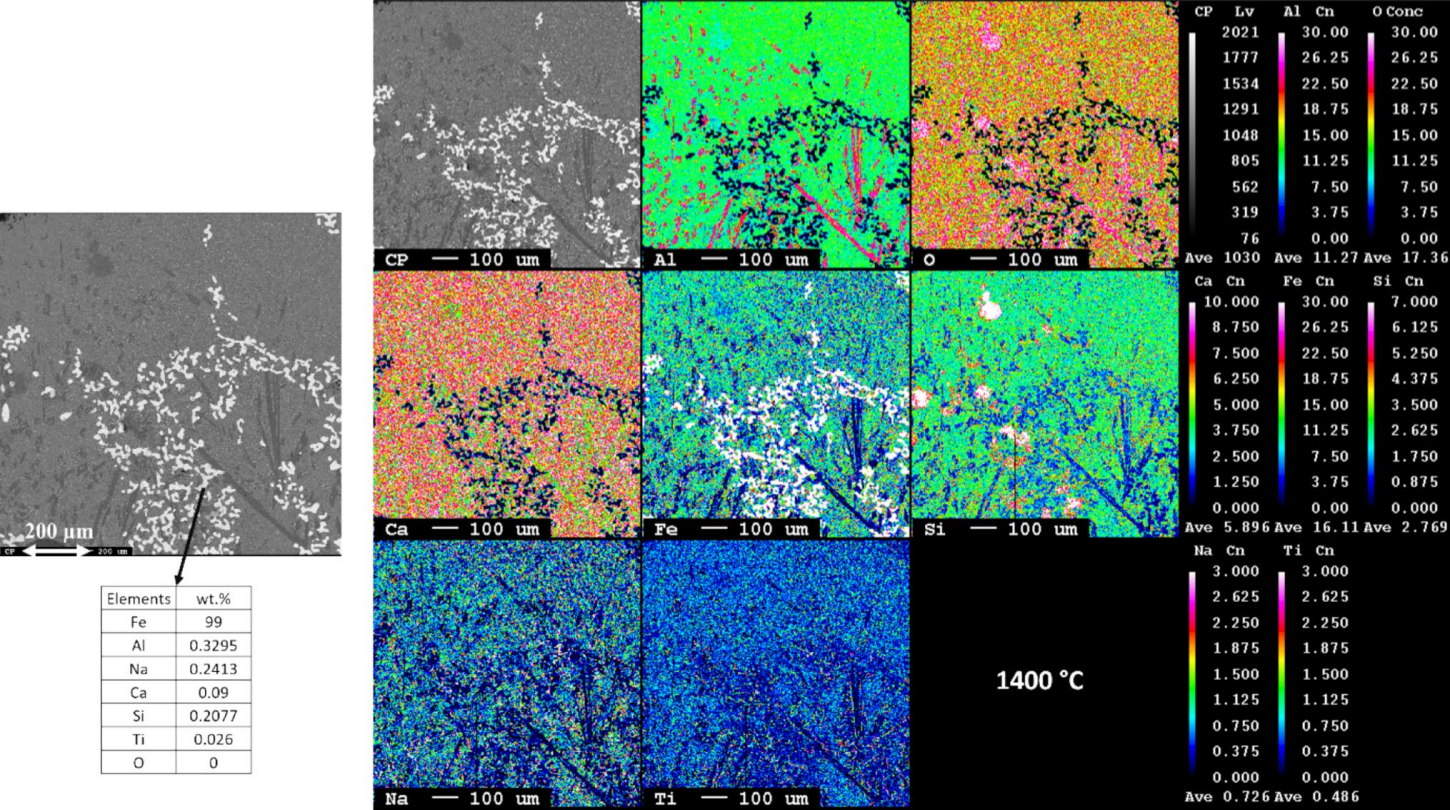
Microstructural and elemental mapping of leaching residue slag smelted at 1400 °C

**Fig. 9:**
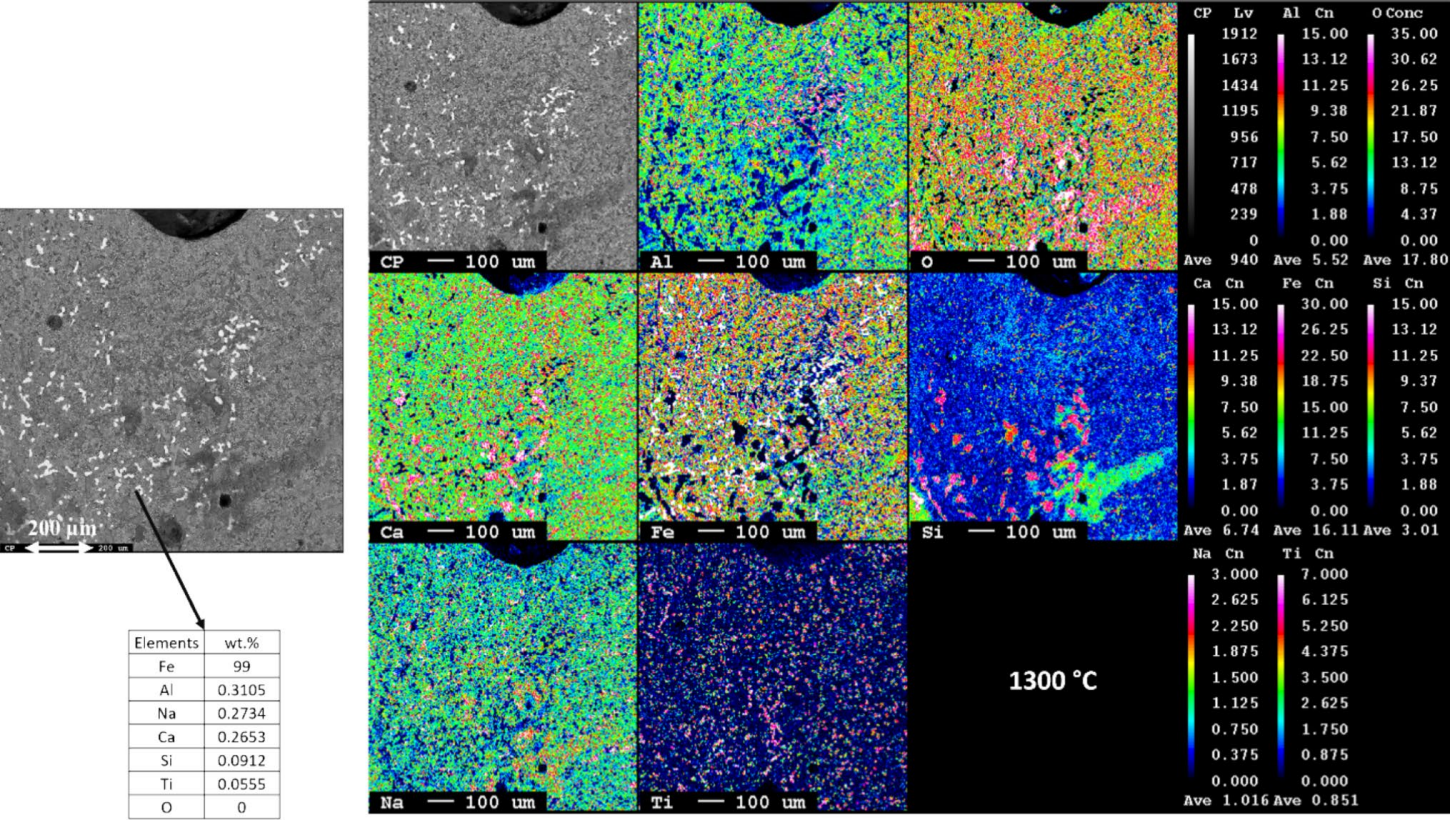
Microstructural and elemental mapping of leaching residue slag smelted at 1300 °C

Figure 7 shows the microstructure and elemental mapping of leaching residue smelted at 1500 °C, revealing three major phases. The bright area corresponds to metallic iron, which contains very small fraction of Al, Na, and Si. However, the metallic iron is present inside the matrix of the slag phase, forming an interconnected network. The dark slag phase mainly composed of Al, Ca, and O. The light dark area is the mixture of Al, Ca, Si, Fe, O, and Ti. Notably, the iron particle size is larger than that in residues smelted at 1400 °C and 1300 °C in Figs. 8 and 9. Obviously, at 1550 °C, almost complete separation of metallic iron from the oxide phase occurred (Fig. 6), while at lower temperatures, the metal separation did not occur completely.

Figure 8 presents the microstructure analysis slag and metal from smelted leaching residue at 1400 °C. The iron particles are uniformly distributed throughout the slag matrix. The iron particles are smaller as compared to those leaching residue smelted at 1500 °C. Moreover, the metallic iron phase contains around 1 wt.% of impurities such as Al, Na, Si, and Ti. In the slag phase, there are two primaries observed. The dark phase is composed of Ca, Al, and O along with that a certain percentage of Fe also present. This Fe may be present in the form of wüstite phase within the stag structure. The light dark matrix comprises a mixture of phases similar to those observed at 1550 °C and 1500 °C temperatures.

The elemental mapping and microstructure of leaching residue smelted at 1300 °C are presented in Fig. 9. Obviously, the iron particles are noticeably finer compared to those smelted at higher temperatures. The composition of the iron particle is similar to the leaching residue smelted at 1400 °C. The dark slag phase contains Ca, Al, and O as the major elements along with Fe, Na, and minor fraction of Ti coexists. The light dark phase is primarily composed of Fe, Ti, and O as the major elements. Based on the weight percentage composition, it can be inferred that the dark areas represent the calcium aluminate phase, while the lighter dark areas are a mixture of the perovskite and wüstite phases. The elemental mapping clearly indicates that iron is distinct and does not overlap with other elements, suggesting that it is separated from the rest of the slag matrix.

**Fig. 10:**
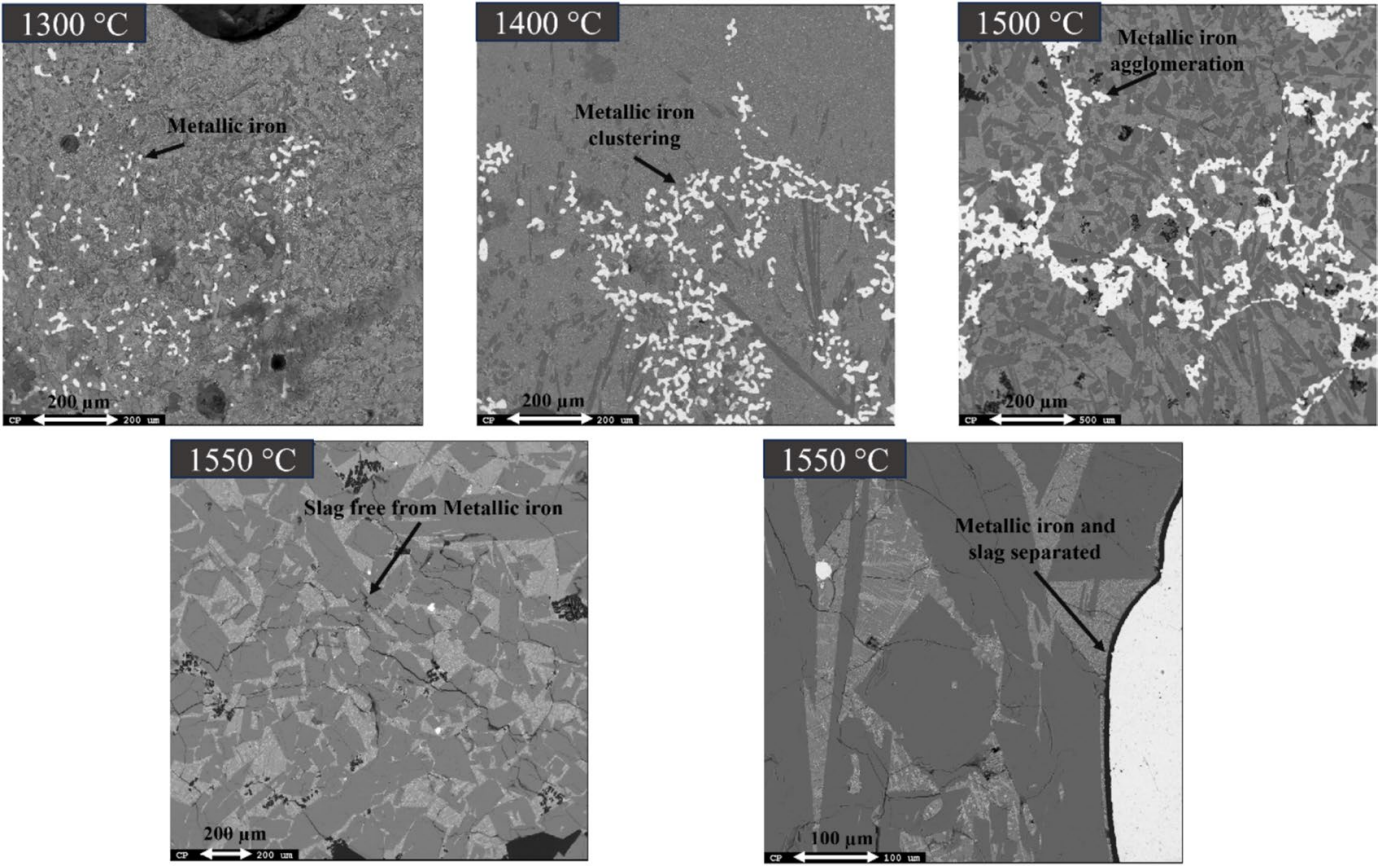
Micrograph of various slag with increasing smelting temperature

The agglomeration of metallic iron with increasing smelting temperature is presented in Fig. 10.

### Elemental Analysis of the Leaching Solution

Table 1 presents the elemental analysis of the leachate produced during the leaching of reduced pellets with a sodium carbonate solution. In addition to Al, a minor portion of Si and S was also transferred to the leaching solution. Based on our previous studies, the transfer of Si into the leaching solution during alkaline leaching can be attributed to the presence of Si in the mayenite lattice [27]. The recovery of alumina is around 62.7%.

**Table 1:** Elemental composition of leachate (ICP) from alkaline leaching of reduced pellets with sodium carbonate solution and metallic iron composition (WDS, EPMA)

	Element	Na	Mg	Al	Si	P	S	K	Ca	Sc	Ti	V	Fe
Leaching	mg/L	38,484	0.5	5791	153	10	366	4.2	3.4	0.0005	0.09	11	0.22
Metallic iron (1550 °C)	wt. %	0.008		0.0006	0.033								99.95

## Discussion

### Mechanism of Self-Hardening During Pelletization

**Fig. 11:**
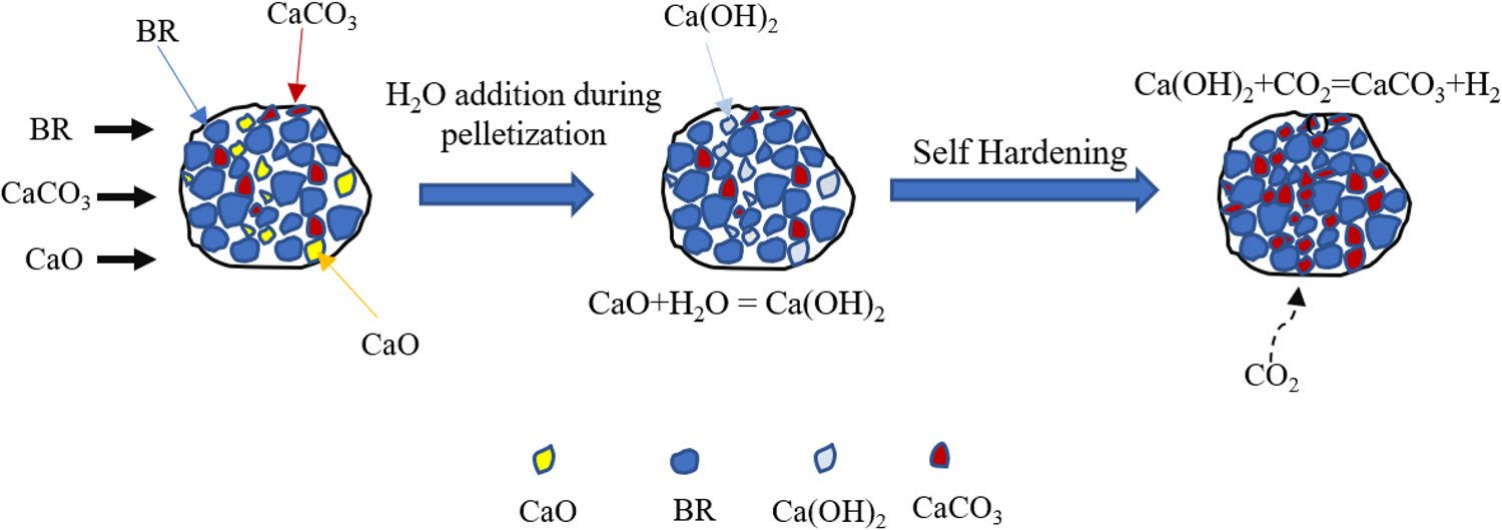
Schematic self-hardening mechanism of bauxite residue and quicklime

Figure 11 illustrates the schematic view of the self-hardening mechanism in pellets composed of bauxite residue, calcite, and quicklime. Both calcite and quick lime are inside in the pellet and between the bauxite residue particles due to the mixture blending. In the pelletization process, the hydration of quicklime occurs in exposure to added water, leading to the formation of calcium hydroxide (Ca(OH)_2_), which potentially acts as a glue between bauxite residue and calcite particles, giving a strength to the green pellets. Overtime, Ca(OH)_2_ reacts with CO_2_ from the atmosphere, resulting in the formation of CaCO_3_. This mechanism contributes to strengthening of the pellets. The conversion of Ca(OH)_2_ to CaCO_3_ is also correlated with the XRD result [28]. Thus, the hardening process of bauxite residue and calcite pellets with variation of CaO are described as below [22]:

1 $$CaO+{H}_{2}O\to Ca{\left(OH\right)}_{2}, \Delta G^\circ =-57.801kJ$$

2 $$Ca{\left(OH\right)}_{2}+C{O}_{2}\to CaC{O}_{3}+{H}_{2}O, \Delta G^\circ =-72.62 kJ$$

Calcite is used to enhance the porosity upon heating, while quick lime facilitates the self-hardening of the pellets. During heat treatment, calcium oxide through its reaction with alumina in the bauxite residue, promoting the formation of calcium aluminate phase.

**Fig. 12:**
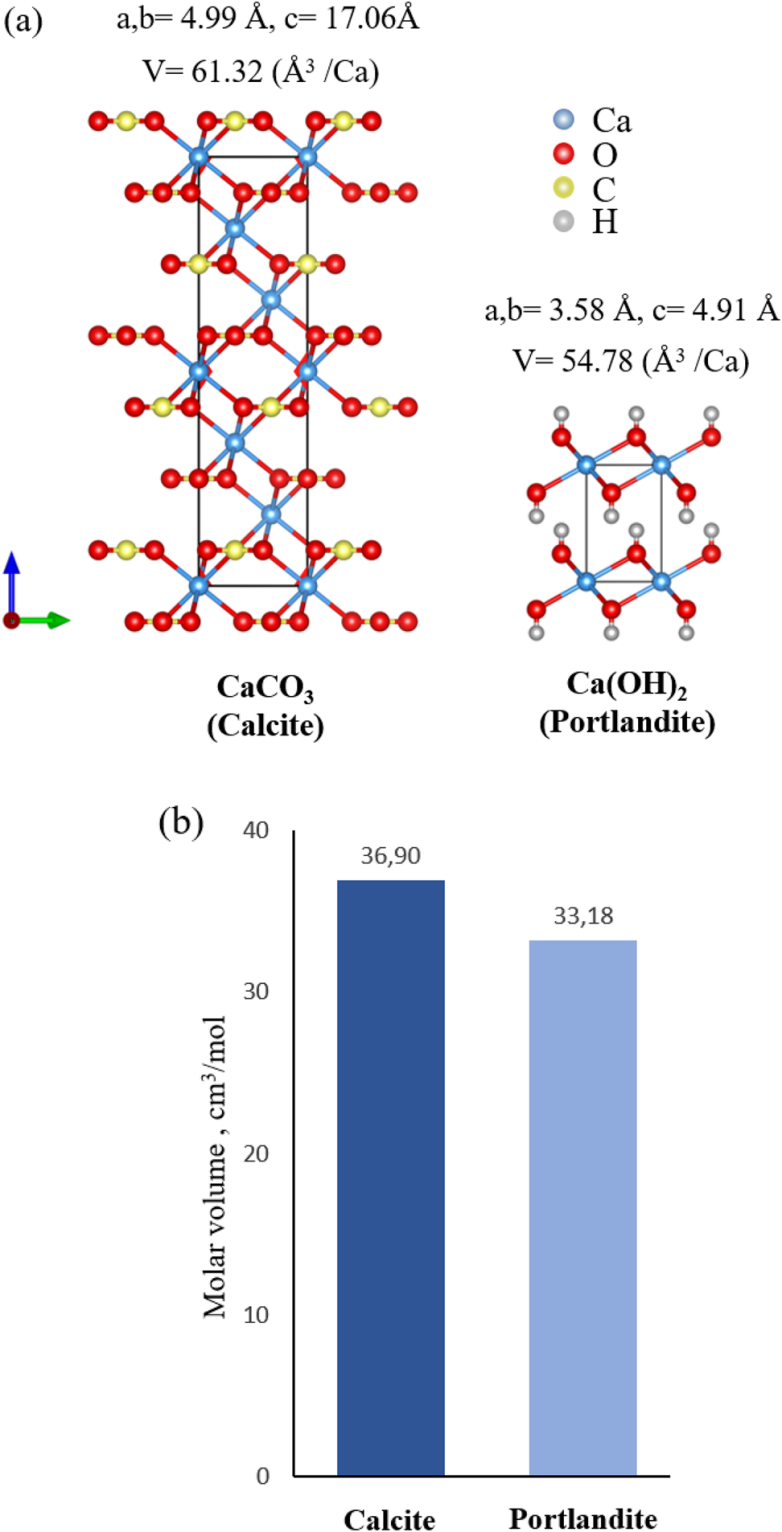
**a** Crystal structure of calcite and portlandite phase with the comparison of unit cell volume necessary for one Ca atom and **b** molar volume of the two phases

Eq. 1 reveals an elevation in volume. In comparing the crystal structures of CaCO_3_ and Ca(OH)_2_, a notable difference is observed in their sizes. As shown in Fig. 12, although both mineral phases belong to the trigonal crystal system, calcite exhibits lattice parameters of a = b = 4.99 Å and c = 17.06 Å, leading to a unit cell volume of 367.92 Å^3^. Given that each unit cell contains six calcium atoms, the volume required for each Ca atom is 61.32 Å^3^. On the other hand, Ca(OH)_2_, with lattice parameter of a = b = 3.58 Å and c = 4.91 Å, has a significantly smaller unit cell of 54.78 Å^3^. Considering its structure, the volume required for one Ca atom is also 53.49 Å^3^. This indicates that the transformation from portlandite to calcite leads to an approximate volume expansion of 11.9%. Similar results can also be obtained by examining the molar volumes of the two phases, revealing a similar expansion of 11.2% in volume [29].

As the volume of CaCO_3_ expands during the carbonation of Ca(OH)_2_, there is a possibility that bauxite residue could be encapsulated within the enlarged volume. This encapsulation of bauxite residue within the expanded volume may lead to the tightening of pellets, potentially enhancing their strength. The tumble index (TI) of the self-hardened pellets is approximately 84.05, with an abrasion index (AI) of around 12.01. In comparison, sintered pellets (sintering temperature 1150 °C, 120 min) with a similar composition have a TI of 85.1 and an AI of 11.8.

### Thermal and Hydrogen Reduction Behavior

**Fig. 13:**
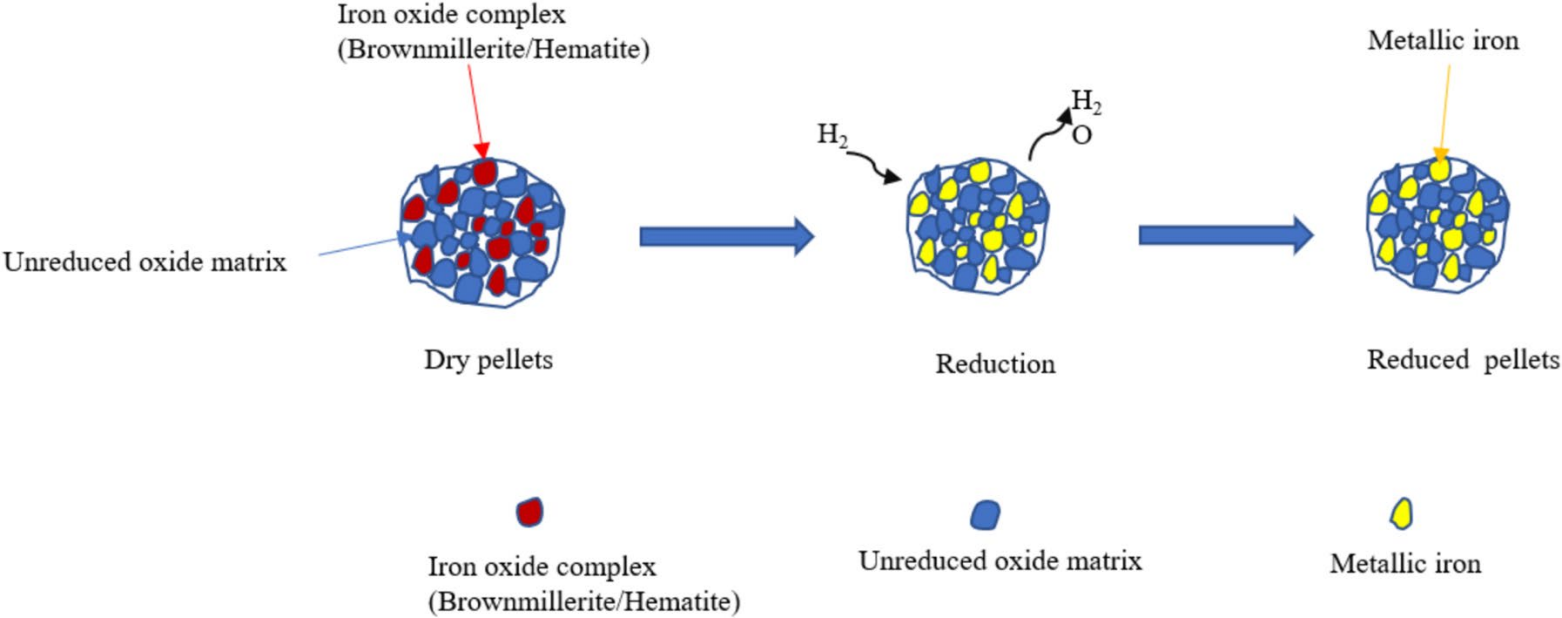
Schematic view of physical reduction of pellets

The schematic diagram of the reduction of dry self-hardened pellets in the presence of hydrogen is shown in Fig. 13. Initially, the iron in these pellets exists as hematite. However, when the pellets are heated to the desired reduction temperature prior to initiating the hydrogen reduction process, a portion of the hematite transforms into brownmillerite (Ca_2_(Fe,Al)_2_O_5_) due to contact with CaO particles, generated from calcite decomposition upon heating [11]. As chemical formula suggests, within the lattice structure of brownmillerite, there is also a minor fraction of Al present. The high-temperature formation of brownmillerite is controlled by mass transport between the solid reactants of CaO, Fe_2_O_3_, and Al_2_O_3_. The latter is formed due to the decomposition of aluminum hydroxides, such as diaspore, upon heating. During heating of the pellets, CaO reacts with Fe_2_O_3_ in the BR, forming Ca_2_Fe_2_O_5_.

When hydrogen is introduced, the reduction mainly takes place through the following steps:

- Step 1: Hydrogen transport from the bulk gas stream to the outer surface of the pellets.
- Step 2: Diffusion of hydrogen to the pellet pores.
- Step 3: Chemical reaction of iron oxide complexes with hydrogen.
- Step 4: Diffusion of product gas (H_2_O) through the product layer and pellet pores to the outer surface of the pellets.
- Step 5: Transport of the product gas to the bulk gas stream.

The reduction reaction of hematite and brownmillerite in the step 3 is as follows:

3 $$F{e}_{2}{O}_{3}+3{H}_{2}\to 2Fe+{3H}_{2}O, \Delta^\circ {G}_{1000^\circ{\rm C} }=-36.31kJ$$

4 $$C{a}_{2}F{e}_{2}{O}_{5}+3{H}_{2}\to CaO+2Fe+{3H}_{2}O ,\Delta^\circ {G}_{1000^\circ{\rm C} }=27.39kJ$$

In addition, during the reduction of self-hardened pellets, other major phases including perovskite (CaTiO_3_), larnite (Ca_2_SiO_4_), mayenite (Ca_12_Al_14_O_33_), and metallic iron are also formed. As shown in Reaction 4, the free energy of the reaction at 1000 °C is positive at pH_2_/pH_2_O = 1. However, in the actual system, pH_2_/pH_2_O is much higher than 1, shifting the equilibrium toward the right. These phases remain stable at a reduction temperature of 1000 °C. The Gibbs free energy of the reactions is presented below. From our previous work, it was found that formation of mayenite is more favorable than calcium titanate and calcium silicate during hydrogen reduction [25].

5 $$CaO+Ti{O}_{2}\to CaTi{O}_{3,}\Delta {G^\circ }_{1000^\circ{\rm C} }=-89.80kJ$$

6 $$2CaO+Si{O}_{2}\to C{a}_{2}Si{O}_{4},\Delta {G^\circ }_{1000^\circ{\rm C} }=-131.24kJ$$

7 $$12CaO+7A{l}_{2}{O}_{3}\to C{a}_{12}A{l}_{14}{O}_{33}\Delta {G^\circ }_{1000^\circ{\rm C} }=-343.92kJ$$

### Mechanism of Alkaline Leaching

**Fig. 14:**
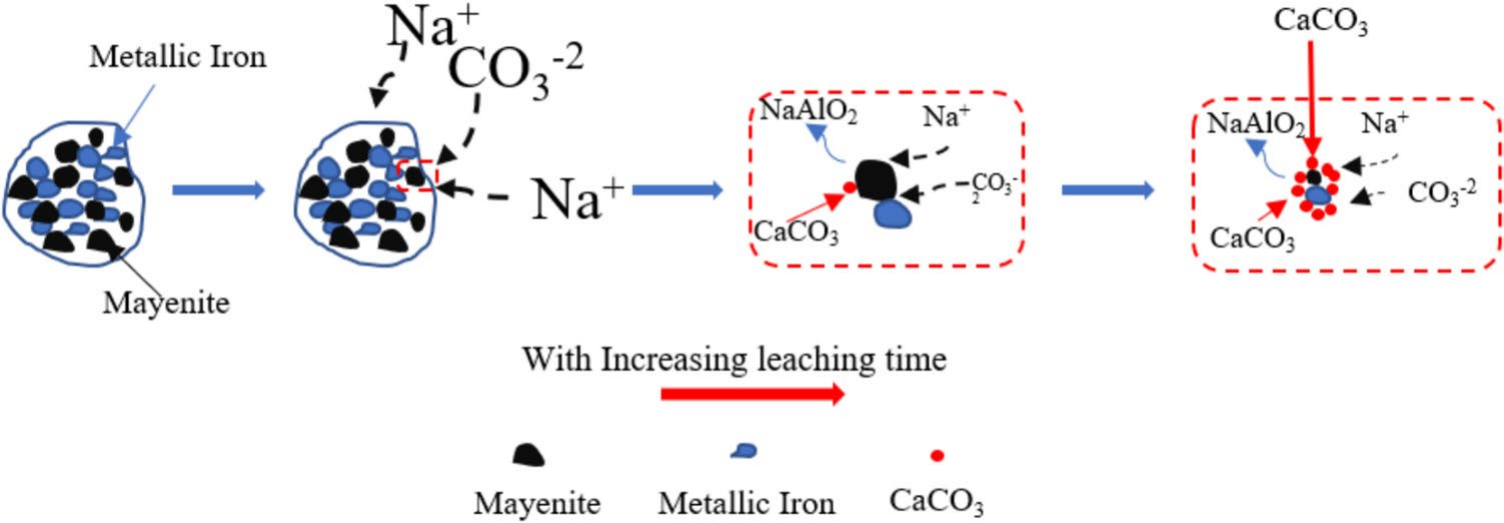
Schematic view of alkaline leaching of mayenite

As shown by the XRD results in Fig. 4, metallic iron and mayenite are identified as the primary phases in the reduced pellets. The alkaline leaching of the reduced pellets aims to recover alumina by converting the mayenite phase to sodium aluminate (NaAlO_2_) solution through the reaction (8). The schematic representation of mayenite leaching with Na_2_CO_3_ is illustrated in Fig. 14. During the alkaline leaching process, the dissolved Na_2_CO_3_ breaking down into ions such as Na^+^ and CO_3_^2−^. The Na^+^ ions react with mayenite, leading to the formation of NaAlO_2_ in solution, while the Ca in the mayenite reacts with CO_3_^2−^ to form calcium carbonate (CaCO_3_). As the leaching time increases, CaCO_3_ accumulates around the unreacted mayenite particles, slowing down the kinetics of the process [30]. Additionally, since iron particle size is smaller, during the leaching process, the produced CaCO_3_ may also form a layer around metallic iron particle which also hinders effective physical separation process. Consequently, the leaching residue mainly consists of metallic iron, calcite, and unleached aluminate phase, perovskite, and larnite; however, iron and calcite are the predominant phases.

8 $$C{a}_{12}A{l}_{14}{O}_{33}+12N{a}_{2}C{O}_{3}+{5H}_{2}O \to 14NaAl{O}_{2}+10NaOH+12CaC{O}_{3}+{5H}_{2}O ,\Delta {G^\circ }_{60^\circ{\rm C} }=-73.08 kJ$$

The XRD spectrum of the leaching residue in Fig. 4 shows that in the applied leaching conditions, metallic Fe is inert and is not oxidized. On the other hand, mayenite has been leached during the process yielding calcite and alumina dissolved in the leachate.

### Leaching Residue Smelting

**Fig. 15:**
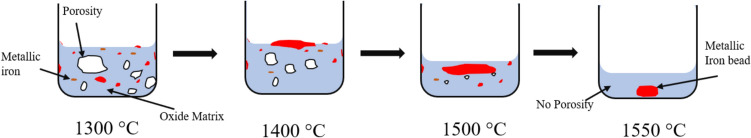
Schematic view of the smelting behavior of leaching residue at different temperatures based on experimental observation

The smelting process of leaching residue effectively separates the metallic iron from other oxides. As schematically shown in Fig. 15, with increasing melting temperature, the metallic iron particles agglomerates. At 1550 °C, the iron particles coalesce to form iron nuggets. At a smelting temperature of 1300 °C, the leaching residue achieved only partial melting without the cohesion of iron particles and the oxide matrix. This led to an increased fraction of porosity and widespread distribution of iron particles throughout. However, as the smelting temperature was raised, porosity decreased, and iron particles began to agglomerate. The loss of porosity, agglomeration of iron particles, and accumulation of oxides were not substantial until the temperature exceeded 1500 °C. A significant amount of calcite is present in the leaching residue, and during the melting process, this calcite decomposes into CaO and carbon dioxide. This phenomenon yields some porosity evolution in the sample. The above microstructures (Fig. 15) indicate that this type of porosity has been lost upon heating to temperatures above 1300 °C. Regarding this, we may conclude that the majority of the oxide portion of pellet has gotten molten state. However, the obtained microstructures indicate that the growth of Fe droplets and their separation were not significant below 1500 °C. Lower surface tension at high temperatures reduces the energy barrier for particles merging, promoting sintering and growth. As surface energy decreases, particles are more likely to coalescence. Smaller particles have a higher surface area-to-volume ratio due to their curvature, which decreases as size increases and correspondingly the surface energy decreases. The produced iron can be utilized in foundries and steelmaking applications.

This phenomenon may be attributed to the higher viscosity of the melt pool at lower temperatures. Moreover, at lower melting temperatures, the higher viscosity impedes the coalescence of iron particles, causing them to remain distributed throughout the oxide matrix.

**Fig. 16:**
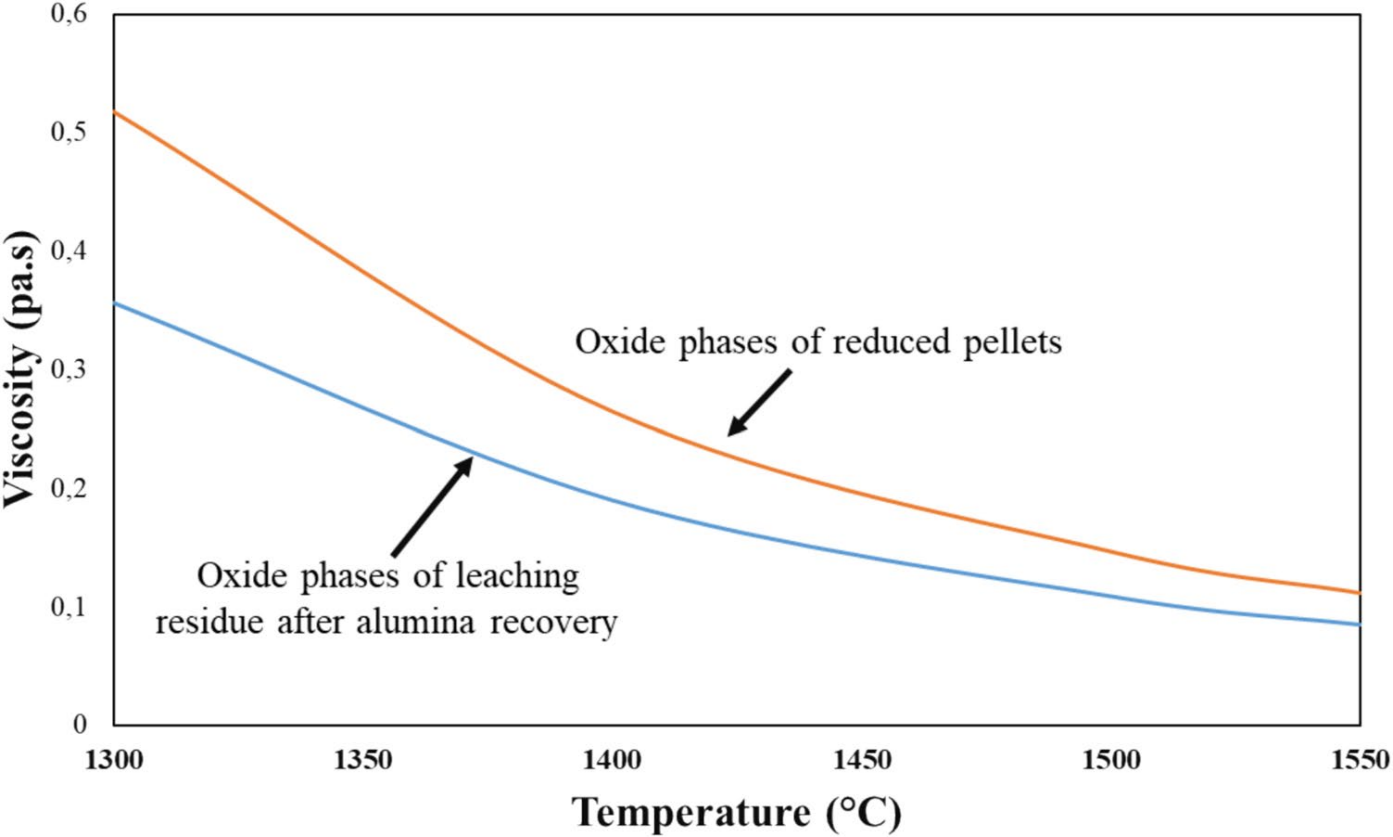
Viscosity of leaching residue slag calculated by FactSage 8.1 with temperature

The viscosity of the leaching residue comprising major oxide components such as CaO, TiO_2_, SiO_2_, and Al_2_O_3_ was calculated by using thermodynamics software FactSage 8.1, as shown in Fig. 16. The Na_2_O was excluded from the calculation due to its higher vapor pressure and susceptibility to loss at higher temperatures, which was confirmed from our previous work [25]. Thus, the calculated viscosity was approximately 0.35 poise at 1300 °C and decreases to 0.05 poise at 1550 °C. It is worth mentioning that although the viscosity calculation was based on the assumption that the slag is in a liquid phase, the calculated viscosity trend still representatively reflects the rheological behavior of the slag during the smelting process.

During the leaching residue smelting process, it was observed that the unleached aluminate phase reacts with calcium oxide to form various calcium aluminate phases. Therefore, both krotite and gehlenite phases are produced. According to the free energy of formation for the aluminate phases, gehlenite exhibits a higher free energy of formation. As listed in the following reaction equations, CaAl_2_O_4_ has a lower Gibbs free energy of formation compared to CaAl_4_O_7_. Despite this, the major calcium aluminate phases identified in the XRD analysis are predominantly CaAl_4_O_7_. This observation suggests that factors beyond just Gibbs free energy, such as reaction kinetics and the deviation of nonstandard conditions, might play a significant role.

9 $$2CaO+A{l}_{2}{O}_{3}\to C{a}_{2}A{l}_{2}{O}_{5}$$

10 $$CaO+2A{l}_{2}{O}_{3}\to CaA{l}_{4}{O}_{7} \Delta {G^\circ }_{\left(1300-1550\right)^\circ{\rm C} }=-\left(66.4-72.99\right)kJ$$

11 $$CaO+A{l}_{2}{O}_{3}\to CaA{l}_{2}{O}_{4,} \Delta {G^\circ }_{\left(1300-1550\right)^\circ{\rm C} }=-\left(49.07-53.75\right)kJ$$

12 $$2CaO+A{l}_{2}{O}_{3}+Si{O}_{2}\to C{a}_{2}A{l}_{2}Si{O}_{7}, \Delta {G^\circ }_{\left(1300-1550\right)^\circ{\rm C} }=-\left(172.23-179.49\right)kJ$$

### Slag Composition as Per FactSage Equilibrium Cooling

Thermodynamic phase calculations for leaching residue slag smelted at various temperatures were performed using FactSage 8.1. These calculations were conducted using the equilibrium module with equilibrium cooling. Four major oxides, namely CaO, TiO_2_, Al_2_O_3_, and SiO_2_, were included in the calculation. Na_2_O was excluded due to its evaporation at high temperatures, as it was not detected in XRD and SEM microscopy analyses.

**Fig. 17:**
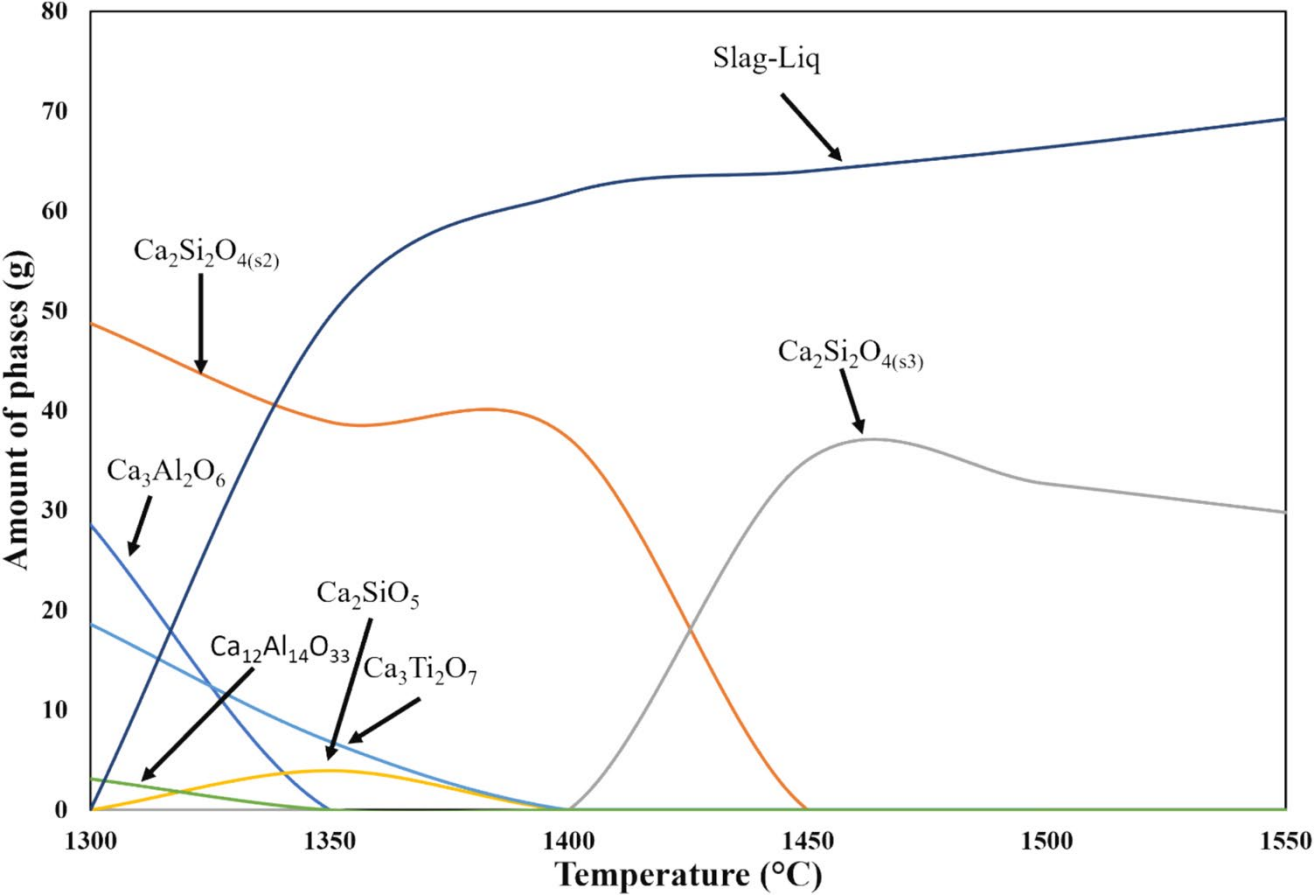
Phase equilibria of the smelted leaching residue at various temperatures, calculated by using FactSage 8.1 equilibrium module

As presented in Fig. 17, the phases formed during equilibrium cooling includes Ca_3_Al_2_O_6_, Ca_2_SiO_4_(s_2_,s_3_), Ca_3_Ti_2_O_7_, Ca_3_SiO_5_, Ca_3_Ti_2_O_7_, Ca_12_Al_14_O_33_ , and slag liquid. The calculated slag phase evolution with varying temperature is shown in Fig. 16. The major phase formed during the melting of slag at 1550 °C is liquid slag and Ca_2_SiO_4_(s_3_). Similar phases with varying amount were observed at 1500 °C smelted slag. Slag melting at 1400 °C, Ca_2_SiO_4_, and slag liquid are the major phases. However, at 1300 °C, all the phases are existed in the solid phase, including Ca_3_Al_2_O_6_, Ca_2_SiO_4_, Ca_3_Ti_2_O_7_, and Ca_12_Al_14_O_33_. According to FactSage calculation, 1341.1 °C marks the final temperature for the disappearance of slag during cooling.

**Fig. 18:**
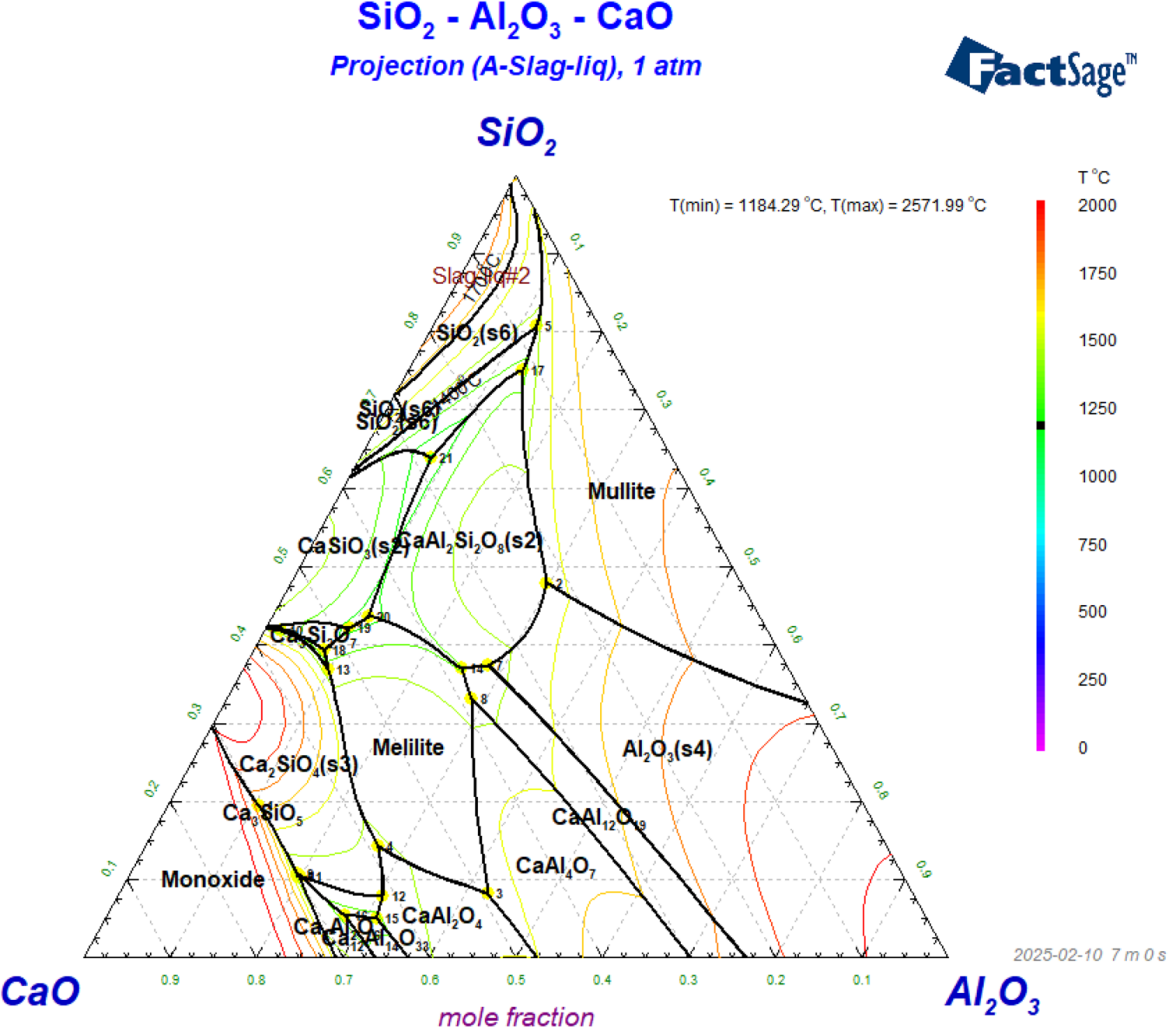
Isothermal projection of CaO–Al_2_O_3_–SiO_2_ system (FactSage 8.3)

As per the XRD analysis, gehlenite (Ca_2_Al_2_SiO_7_), calcium aluminates (Ca_2_Al_2_O_5_, CaAl_2_O_4_, CaAl_4_O_7_), and perovskite (CaTiO_3_) are the major phases found in the produced slags. However, Ca_2_SiO_4_, Ca_3_Al_2_O_6_, and Ca_3_Ti_2_O_7_ are the major phases found in the equilibrium calculation. At higher temperatures, a (Ca, Ti)-based solid solution, which is rich in Ca_3_Ti_2_O_7_ and Ca_3_Ti_2_O_6_, forms instead of the experimentally determined perovskite. The deviation between experimental result and FactSage calculation may be due to kinetics and faster cooling. According to the XRD results at 1300 °C, mayenite phase was found and it is also correlated with the FactSage calculation. As shown in Fig. 18, the equilibrium diagram of CaO–Al_2_O_3_–SiO_2_, the phase CaAl_2_O_4_ is stable at higher smelting temperatures.

## Conclusion

The present work investigated a novel approach for the effective utilization and valorization of bauxite residue for iron and alumina production. The integrated process involves hydrogen reduction, alkaline leaching, and smelting of the leaching residue. The conclusions derived from the experimental work are as follows:

1. The self-hardening process of bauxite residue calcite pellets involves the cementing nature of quicklime. Quicklime absorbs water to form calcium hydroxide, which further absorbs CO_2_ from the atmosphere, leading to the formation of calcium carbonate. The increase in volume may encapsulate the bauxite residue, contributing to the strengthening of the pellets.
2. During the reduction of self-hardened pellets, iron oxide complexes were effectively converted to metallic iron at 1000 °C and 120 min of reduction time.
3. Alumina was extracted from the reduced pellets via alkaline leaching, facilitated by the presence of the mayenite phase. The remaining alumina in the leaching residue is primarily attributed to the formation of indissoluble gehlenite during the prior heating process, coupled with the hindered kinetics as the development of a dense calcium carbonate layer.
4. Smelting of the leaching residue for iron removal is effective above 1500 °C, attributed to higher iron purity and the presence of higher melting oxide phases in the leaching residue.
5. The smelting process of the leaching residue experiences increased viscosity due to high-melting-point oxides, which obstructs the agglomeration of metallic iron. As the smelting temperature increases, viscosity decreases, allowing for iron agglomeration to occur.
6. The smelted slag is primarily composed of calcium oxide, with a fraction of alumina and silica, making it a suitable material for construction applications.

There are some following advantage and limitations of the process presented here.

One key advantage is the use of CO₂ in precipitation and the potential for Ca recycling by integrating the high-CaO slag into the bauxite residue feed during pelletizing. This approach helps minimize CaO consumption.

However, the process has notable limitations:

The high production cost of hydrogen reduction, along with its significant energy requirements, presents a major challenge. Additional energy consumption occurs during the smelting of the leaching residue, further increasing the overall process cost.

## Figures and Tables

**Fig. 19:**
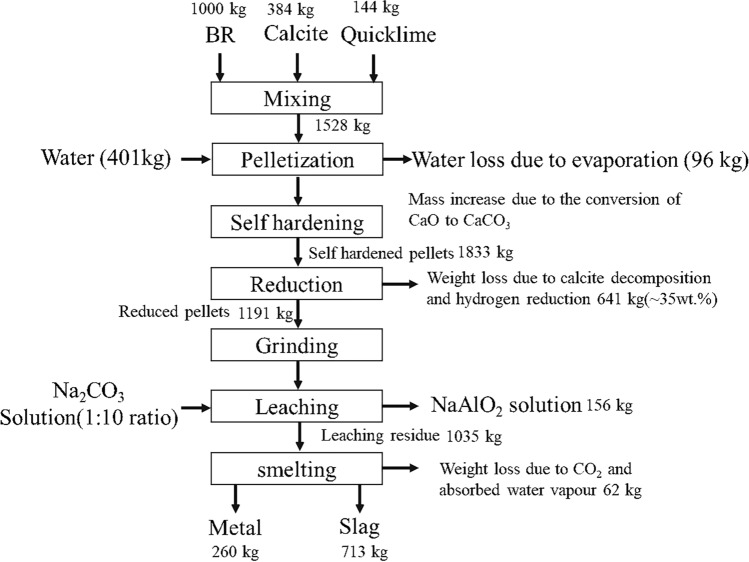
Mass balance of the proposed process

## Appendix A1

See Table 2.

**Table 2:** Chemical analysis of the bauxite residue, self-hardened pellets, reduced pellets, leaching residue, and slag produced at 1550 °C (wt.% is normalized with respect to loss of ignition)

wt.%	Bauxite residue	Self-hardened pellets	Reduced pellets	Leaching residue	Slag (1550 °C)
SiO_2_	7.43	5.61	7.34	9.03	12.06
MnO	0.09	0.05	0.03	0.05	0.06
P_2_O_5_	0.12	0.07	0.11	0.12	0.16
SO_3_	1.04	0.60	1.00	0.65	0.86
Cr_2_O_3_	0.22	0.23	0.28	0.29	0.39
NiO	0.00	0.12	0.08	0.07	0.09
TiO_2_	5.79	4.09	5.03	5.51	7.36
V_2_O_5_	0.14	0.13	0.00	0.15	0.20
Al_2_O_3_	25.37	17.41	19.18	7.04	9.40
CaO	10.50	36.15	40.27	46.44	62.03
Fe_2_O_3_/Fe	44.72	32.98	23.08(Fe)	25.13(Fe)	0.00
ZrO_2_	0.00	0.12	0.16	0.00	0.00
MgO	1.20	0.56	0.62	0.82	1.10
Na_2_O	3.28	1.68	2.58	4.50	6.02
K_2_O	0.10	0.07	0.04	0.00	0.00
ZrO_2_	0.00	0.12	0.16	0.14	0.19
SrO	0.00	0.02	0.02	0.02	0.03
WO_3_	0.00	0.00	0.02	0.04	0.05

## Appendix A2

See Fig. 19.
